# Cultural and psychological dimensions of the superwoman identity among Jordanian women

**DOI:** 10.3389/fpsyg.2026.1676125

**Published:** 2026-02-18

**Authors:** Yasmeen Moshtaq A. Al Issa, Shulin Chen

**Affiliations:** Department of Psychology and Behavioral Sciences, Zhejiang University, Hangzhou, Zhejiang, China

**Keywords:** cultural expectations, ethnography, gender roles, Jordanian women, Superwoman Schema

## Abstract

**Background:**

The Superwoman Schema (SWS) reflects the expectation that women sustain strength, emotional control, and self-sacrifice while managing multiple roles. Although extensively examined in Western contexts, little is known about its relevance in non-Western, patriarchal cultures where motherhood and caregiving form central components of women's social identity.

**Methods:**

Using an ethnographic qualitative design, this study explored how 43 Jordanian women negotiate the Superwoman (SW) identity within sociocultural expectations that equate feminine fulfillment with motherhood. Five focus groups were conducted, and the data were analyzed thematically.

**Results:**

Analysis identified five themes: (1) cultural constructions of the superwoman identity, (2) women manifesting strength, (3) structural barriers and lived struggles, (4) empowered transitions in women's identity, and (5) everyday coping and sustainable wellbeing. Caregiving emerged as the core site for enacting the SW, with maternal sacrifice celebrated yet producing emotional exhaustion, internalized pressure, and diminished wellbeing.

**Conclusion:**

This study is among the first to use the SWS framework in Jordan, a conservative and patriarchal context. The findings reveal culturally specific manifestations of the SW identity, where resilience is a source of pride but also linked to emotional exhaustion and the psychological costs of suppression. Younger women's emerging emphasis on self-care and boundaries signals a shift toward more sustainable models of empowerment. The study's significance lies in advancing cross-cultural understanding of gendered stress and providing evidence to inform culturally sensitive mental health interventions, community programs, and policy that promote women's wellbeing while respecting cultural and religious values.

## 1 Introduction

The Superwoman (SW) and Strong Independent Woman (SIW) identities have gained increasing visibility in contemporary discourse, particularly through digital media and feminist rhetoric that emphasize empowerment, resilience, and self-sufficiency (Dove-Viebahn, 2023; Nelson et al., 2016), yet these ideals embody a profound paradox: they affirm women's agency and strength while simultaneously imposing expectations of constant perseverance, emotional restraint, and self-sacrifice (Woods-Giscombé, 2010; Nicolson, 2003; Geyton et al., 2022; Beauboeuf-Lafontant, 2007). This tension is illustrated by the Superwoman Schema (SWS) (Woods-Giscombé, 2010), originally developed to describe the experiences of African American women negotiating intersecting burdens, which highlights how the internalization of strength as a moral and cultural imperative can both protect against and perpetuate psychological distress. Similar paradoxes unfold in Middle Eastern Muslim patriarchal societies (Pappé, 2014), where the widespread social media influence of the SW identity (Rosenmann et al., 2016; Judijanto, 2024) clashes with conservative, faith-based norms idealizing women's patience (ṣabr), modesty, and self-sacrifice for family cohesion (Haque, 2025; Pappé, 2014). Consequently, many women find themselves caught between modern ideals of independence and enduring cultural expectations of obedience and emotional restraint (Narayan, 2018), confirming the SWS framework's value as a lens to examine how imported models of female empowerment, when filtered through religious and patriarchal systems, can create unique forms of psychological tension and self-silencing in Middle Eastern contexts.

The SWS framework delineates five interrelated dimensions: the obligation to present an image of strength, the suppression of emotions, resistance to vulnerability, the determination to succeed despite limited resources, and the prioritization of caregiving over self-care (Woods-Giscombé, 2010; Woods-Giscombe et al., 2019). Conceptualized originally to capture the psychosocial experiences of African American women, the SWS provides a culturally grounded model that links internalized ideals of strength, emotional restraint, and caregiving to both resilience and psychological strain. Rooted in a historical legacy of racial and gender oppression, the model illustrates how sociocultural expectations to remain strong, conceal distress, and prioritize others' needs operate as adaptive coping strategies for survival, yet paradoxically contribute to chronic stress and health disparities.

In adapting this framework to a Middle Eastern, patriarchal context, this paper proposes a reconceptualization of the antecedents of the SWS to reflect the influence of religious moral codes, collectivist values, and gendered family roles that similarly valorize endurance, obedience, and self-sacrifice as feminine virtues. While Woods-Giscombé's model emphasized racialized survival stressors, this adaptation seeks to explore comparable tensions between traditional expectations of patience (ṣabr) and emerging ideals of independence shaped by globalization and modernity. This adapted framework is therefore designed to preserve the core SWS dimensions (strength, emotional restraint, and caregiving) but situates them within the cultural logics of honor, modesty, and faith. In this study, we aimed to illustrate how global discourses of female empowerment intersect with local gender ideologies to shape women's psychological wellbeing.

Over time, the SWS has been refined and validated through empirical and cross-disciplinary research. Psychometric tools such as the Giscombe Superwoman Schema Scale (Woods-Giscombe et al., 2019) have confirmed its multidimensional structure, while studies have linked the construct to stress, coping, stigma, and health disparities (Beauboeuf-Lafontant, 2003; Black and Peacock, 2011; Settles et al., 2008). These theoretically grounded models provide a structured lens for examining how cultural expectations of strength, emotional suppression, caregiving, and self-sacrifice shape women's psychological wellbeing (Beauboeuf-Lafontant, 2003; Black and Peacock, 2011; Woods-Giscombé, 2010). Yet, most of this research has focused on Western and White-dominant contexts, leaving its manifestations in other cultural settings largely unexplored (Tlaiss, 2015; Moghadam, 2004; Gerner, 2020).

Jordan, as a predominantly Muslim and Arab country, offers a distinctive context for expanding SWS exploration. Historically, conservative cultural and religious norms shaped gender roles, with women expected to manage domestic responsibilities and caregiving, while men assumed the role of financial providers (Sonbol, 2003; Leslie et al., 2015; Murphy, 2002; Fitria, 2025). Despite significant progress in women's access to education and employment, gender disparities remain entrenched (El Khawand, 2025). Men continue to enjoy preferential advantages in the labor market, often receiving greater opportunities and privileges on the basis of gender (Miles, 2002). Within families, men typically retain broader freedoms and authority compared to women (Dankwa, 2018), and resistance to women's participation in political life persists, with some men opposing their involvement in decision-making roles (Wayan and Nyoman, 2020). Although the Jordanian government has introduced policies aimed at empowering women and challenging stereotypes (Aloun, 2024), progress has been limited. According to the Department of Statistics (2024)^1^, women constitute 47.1% of the total population, with 60.7% in the working-age group (15–64 years). Yet, in 2024 their unemployment rate reached 32.9%, nearly twice that of men (18.2%). These structural inequalities in labor participation reinforce the psychological and social pressures identified in this study, as women must navigate cultural expectations of caregiving and obedience while facing limited economic opportunities and barriers to independence.

Research on work–family dynamics consistently demonstrates that family expectations and parental demands play a substantial role in shaping women's experiences of work–family conflict (Noor, 2004). Prior studies show that women disproportionately shoulder the responsibility for caregiving and household management (Cinamon and Rich, 2002), and such gendered expectations heighten the strain of balancing professional and domestic obligations. Work–family conflict has been associated with numerous adverse outcomes, including life strain, emotional exhaustion, and diminished psychological wellbeing (Hagqvist et al., 2017; Prasiska et al., 2024), suggesting a potential pathway through which family pressures contribute to broader life stress. Evidence also highlights the protective role of spousal support in mitigating the effects of parental and familial demands by facilitating a more equitable distribution of domestic responsibilities (Matsui et al., 1995).

While expanded access to education and employment has created new opportunities, deeply entrenched norms continue to assign women the primary responsibility for caregiving and domestic labor (Boustati, 2020; Mehtap et al., 2016; Sidani, 2005; Moghadam, 2004). This dual burden compels women to excel professionally while maintaining traditional gender roles, fostering a constant need to prove their competence as both devoted caregivers and successful workers (Koburtay et al., 2020). Yet, little empirical research has examined how Jordanian women negotiate these tensions, leaving important gaps in understanding the psychological and emotional dimensions of the SWS in Arab societies.

To our knowledge, this is the first study to investigate the SWS in the Jordanian context. Specifically, it examines how women conceptualize and embody the SW identity within a patriarchal society, and the socio-cultural and systemic factors shaping their lived experiences. It further explores the psychological and emotional implications of adopting this identity, highlighting both its empowering and burdensome aspects. By extending discourse beyond its Western-centric focus, this study contributes to a more comprehensive understanding of the cross-cultural relevance and impact of the SWS. Moreover, by foregrounding the challenges associated with these expectations, it underscores the need for inclusive discussions on gender roles, societal norms, and policy implications in Arab societies.

Research questions:

1. How do Jordanian women define SW identity?
2. How is the SW identity perceived within Jordanian society?
3. What emotional responses (accepting or rejecting) does it evoke among women?
4. How do women understand generational differences in gender roles, especially in relation to the increased exposure to global ideals of autonomy and independence?

## 2 Materials and methods

### 2.1 Study design

This study employed an ethnographic qualitative study design to examine Jordanian women's perceptions and lived experiences of the SWS. The ethnographic approach (Case et al., 2014; Simanjuntak and Hendriani, 2022) enabled the exploration of how Jordanian women internalize, negotiate, and resist the SWS in ways deeply embedded within cultural, religious, and gender dynamics. By capturing nuanced descriptions of participants' lived experiences, this approach facilitated a deeper understanding of the psychological and sociocultural dimensions underpinning the SW identity in Jordanian society.

All participants received detailed information about the study's purpose, the topics to be discussed during the focus groups, and any potential risks associated with sharing personal experiences. They were assured of confidentiality, informed of their right to withdraw at any point without consequence, and asked to provide written informed consent before participation. To minimize potential discomfort, participants were reminded that they could decline to answer any question or withdraw from participation at any time. In addition, contact information was available if any emotional distress occurred. Participants received modest compensation in recognition of their time and contribution to the study.

### 2.2 Settings and procedure

Five audio-recorded focus groups were conducted in person, in Arabic, and lasted between 90 and 120 min. A semi-structured format was employed, guided by a standardized set of open-ended questions that ensured comparability across groups (see Supplementary material for the interview guide), while allowing flexibility for probing and the emergence of participant-driven themes. A trained moderator facilitated the discussions, fostering inclusive dialogue and encouraging contributions from less vocal participants to ensure balanced engagement, while ensuring that participants' ideas and perspectives were freely expressed, represented accurately, and without any influence. The focus groups explored participants' views on the SW identity, including its associated societal roles, their emotional implications of these roles. To capture diverse perspectives, the sessions were organized in collaboration with the Ministry of Education and non-governmental organizations across different regions of the country, and local permissions were obtained.

### 2.3 Participants

The study includes 43 Jordanian women with lived experiences of managing multiple social roles. Participants were recruited through snowball sampling and targeted invitations to ensure diversity across age, marital and parental status, employment, and socioeconomic levels. Participants were eligible if they met the following criteria: (a) Jordanian women who were raised in Jordan, and (b) expressed a willingness to share their lived experiences regarding gender roles. Participants were excluded if they were: (a) under the legal marriage age (18 years), (b) non-Jordanian, or (c) having a prior diagnosis of a mental health disorder.

The study included participants who ranged in age from 23 to 58 years and represented varied marital statuses, educational levels, and employment conditions. Data collection continued until thematic saturation was achieved, which was confirmed when subsequent group discussion yielded no new theoretical insights. Table 1 presents the demographic composition of the focus groups.

**Table 1:** Demographic composition of participants across the five focus groups.

**Focus groups**	**Number of participants**	**Age range (year)**	**Marital status**	**Education level**	**Employment status**
FG 1	8	23-58	5 Married, 3 Singles	4 Graduates, 4 Undergraduates	6 Employed, 2 Unemployed
FG 2	8	26-46	5 Married, 2 Singles, 1 Divorced	2 Graduates, 5 Undergraduates, 1 High school	4 Employed, 4 Unemployed
FG 3	10	27-55	4 Married, 3 Singles, 3 Widowed	1 Graduate, 4 Undergraduates, 5 High school	5 Employed, 5 Unemployed
FG 4	10	32-47	6 Married, 4 Singles	3 Graduates, 7 Undergraduates	9 Employed, 1 Unemployed
FG 5	7	26-45	5 Married, 2 Singles	2 Graduates, 4 Undergraduates, 1 High school	7 Employed

FG, Focus Group.

### 2.4 Reflexivity

As a Jordanian woman and researcher, my cultural proximity to the participants positioned me as both an insider and observer. This dual role offered deep contextual insight into the social and gendered dynamics shaping the SWS in Jordan. While my shared cultural background fostered trust and openness during data collection, it also required ongoing reflexivity to distinguish participants' voices from my own assumptions and experiences. I remained attentive to how my interpretations were shaped by personal values and professional training. To mitigate potential bias, I engaged in regular memo writing, peer debriefing, and cross-checking with other team members to ensure that participants' narratives were represented with integrity and authenticity.

### 2.5 Data analysis

Data were analyzed using deductive thematic analysis, a qualitative methodology that facilitates the systematic identification, organization, and interpretation of patterns within textual data (Braun and Clarke, 2022; Attride-Stirling, 2001; Dörnyei and Griffee, 2010). This analysis was conducted with the assistance of NVivo 14 software, which enabled efficient coding, categorization, and retrieval of data (Wong, 2008; Richards, 1999).

The thematic analysis followed a structured, multi-step process to ensure a comprehensive and accurate interpretation of the data. After data collection, the first author transcribed all audio recordings. Transcripts were subsequently cross-checked by the research team to verify consistency and uphold data integrity, and then all transcripts were translated into English by a language expert. Once the transcription was completed, the data familiarization phase began, where the researchers and coders thoroughly reviewed the transcripts to gain an initial understanding of participants' responses. This step helped identify areas that required deeper analysis.

Next, during the coding phase, two trained coders who are Jordanians and familiar with the culture, independently and systematically labeled key elements from participants' narratives with descriptive codes, capturing recurring patterns and emerging themes. The initial codes were then compared between coders, with any discrepancies discussed and resolved through consensus. Subsequently, the codes were organized into broader subthemes and overarching themes, interpreted in accordance with the study's objectives. The analytical process explored how participants defined and perceived the SW identity, the emotional responses it elicited, its expression in women's social and familial roles, and the generational shifts shaped by younger women's exposure to global ideals of autonomy. In the subsequent phase, the emerging themes were iteratively reviewed and refined to ensure internal coherence and analytic depth.

Finally, in the interpretation and reporting phase, the findings were analyzed, focusing on the underlying patterns and contradictions within the data. This comprehensive process provided a nuanced understanding of the participants' experiences, capturing both the complexity and richness of the data, and contributing to broader discussions on gender roles, mental health, and sociocultural influences in Jordanian society.

**Figure 1:**
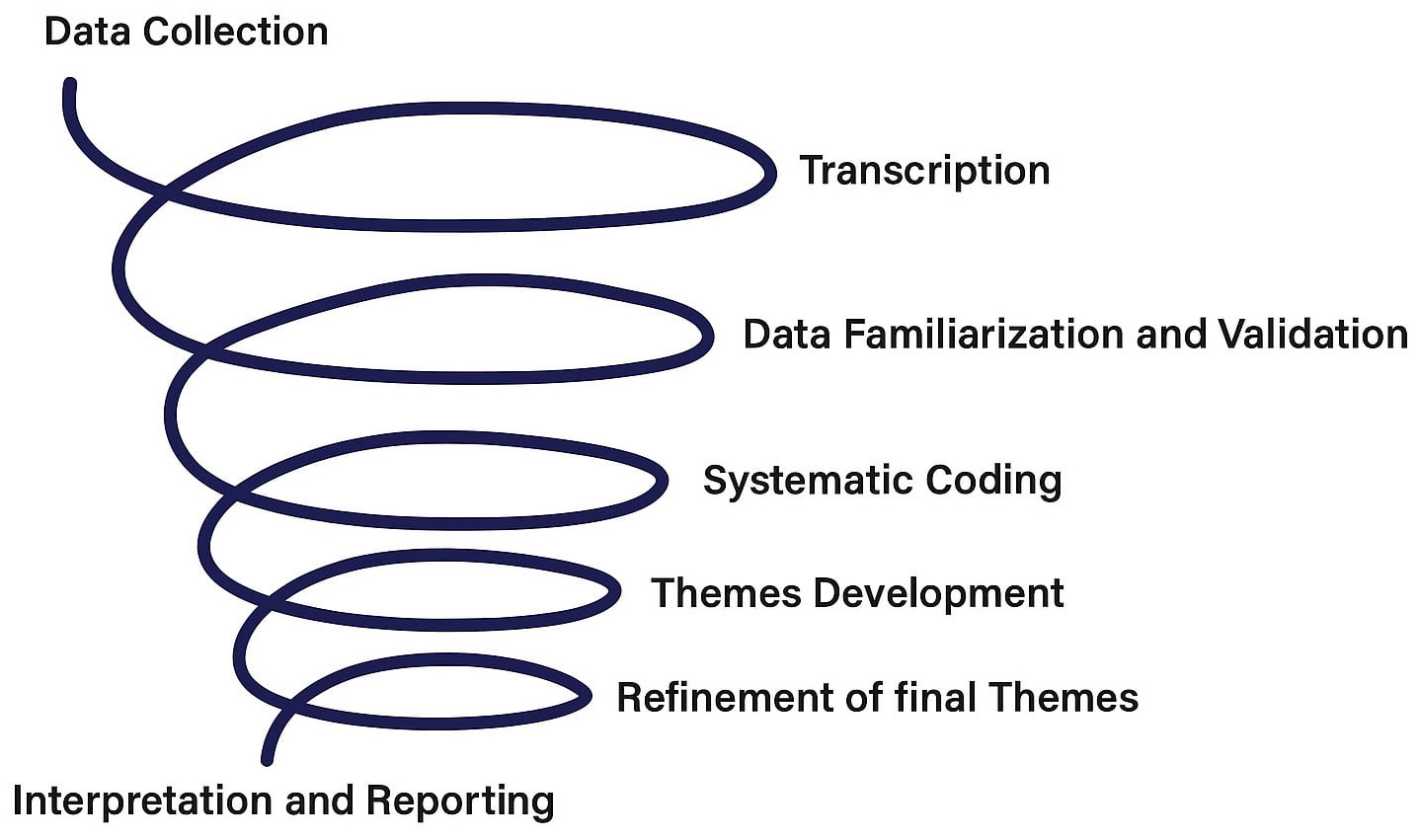
The spiral process of thematic analysis.

The use of NVivo software (Zamawe, 2015) facilitated the organization and retrieval of coded data, enabling systematic comparisons across participants and ensuring analytical rigor. This approach provided a nuanced understanding of how Jordanian women navigate the expectations associated with the SW identity, while ensuring the authenticity of participants' voices by cross-checking the transcripts and key themes with another researcher familiar with Jordanian society and culture (see Figure 1).

## 3 Results

Thematic analysis of the focus group discussions identified five main themes. These were: (1) Cultural Constructions of the SW Identity, (2) Women Manifesting Strength, (3) Structural Barriers and Lived Struggles, (4) Empowered Transitions in Women's Identity, (5) Everyday Coping and Sustainable Wellbeing. Based on the views of the participants, these themes are further divided into 17 subthemes (see Table 2).

**Table 2:** Key themes, subthemes, and subthemes definition derived from the focus group discussions analysis.

**Themes**	**Subthemes**	**Subthemes definition**
Cultural constructions of the superwoman identity	Cultural blueprints	Societal scripts that define women's strength through obedience, endurance, and self-sacrifice, prescribing narrow roles and limiting autonomy.
	Inherited ideals	Family-transmitted beliefs that equate womanhood with caregiving, patience, and self-denial are modeled by older generations.
Women manifesting strength	The silent fighter	Represents women who embody endurance, patience, and perseverance, managing multiple responsibilities with quiet strength while concealing emotional exhaustion beneath composure and resilience.
	Because she is the giver	Captures the moral expectation that women naturally assume caregiving and nurturing roles, positioning caregiving, especially motherhood, as women's primary source of worth, even amid professional strain.
	Who can fulfill the requirements?	Highlights the conditional nature of the SW ideal, showing that strength is variously defined through autonomy, lived experience, or resilience rather than formal education or marital conformity.
	Resisting the identity	Describes women's growing critique of the SW ideal as burdensome and restrictive, as they reject unrealistic expectations of sacrifice and redefine strength around authenticity, agency, and self-care.
Structural barriers and lived struggles	Gendered power and women's autonomy	Describes how patriarchal authority and male guardianship limit women's freedom of choice, mobility, and education, framing obedience as virtue while constraining self-determination and opportunity.
	Inner dispute	Captures women's internal conflict between conforming to cultural expectations of obedience and expressing independent perspectives, leading to emotional strain and self-silencing.
	Dual burdens	Highlights how women shoulder intersecting domestic and professional responsibilities while their achievements remain undervalued or dependent on male approval within family and institutional hierarchies.
	Constrained choices and aspirations	Reflects how structural and relational barriers such as male permission and social norms, restrict women's pursuit of education, career, and autonomy, resulting in deferred or unrealized ambitions.
Empowered transitions in women's identity	Cultivating self-belief	Represents women's redefinition of empowerment as confidence, agency, and goal-driven action, shifting strength from silent endurance to self-determined pursuit of purpose and fulfillment.
	Strategic self-actualization	Describes empowerment as an active, reflective process of personal growth, setting boundaries, balancing ambition with wellbeing, and engaging meaningfully in public and professional life.
	Financial and intellectual autonomy	Highlights independence as a foundation of empowerment, where financial self-sufficiency and critical thinking enable women to act freely, make informed decisions, and negotiate cultural expectations.
	Pursuing wholeness	Frames empowerment as holistic alignment of spiritual, emotional, and practical dimensions, emphasizing inner peace, moral integrity, and resilience as markers of authentic strength.
Everyday coping and sustainable wellbeing	Relational anchors	Emphasizes that resilience and empowerment emerge through connection, mutual support, and shared responsibility, reframing strength as interdependence rather than solitary endurance.
	Grounding practices	Refers to intentional daily routines, such as journaling, reading, or solitude, that help women maintain emotional balance, clarity, and self-regulation amid multiple demands.
	Joyful engagement	Highlights engagement in pleasurable and creative activities as a proactive form of coping that nurtures vitality, self-worth, and relational harmony.

### 3.1 Cultural constructions of the superwoman identity

This theme addresses how cultural values and social norms in Jordan shape the SW identity, embedding expectations of obedience, self-sacrifice, and resilience. While often celebrated as empowering, this identity also generates profound emotional and psychological burdens, constraining women's autonomy and opportunities for growth. Participant accounts reveal a cultural paradox: the qualities revered as “strength” by society are frequently experienced as submission by women themselves.

#### 3.1.1 Cultural blueprints

Cultural scripts have historically positioned the SW identity within a framework of obedience, devotion, and self-sacrifice, channeling women into prescribed roles in education, marriage, and caregiving with minimal space for deviation.

“I consider myself a SW, yet my society does not perceive me as such… the SW is the obedient one… a woman is expected to conform and accept whatever her husband or brother tells her without question. But from another perspective, she is not a SW; she is simply a woman who has surrendered.” (48 years, married, employed).

This narrative illustrates a central paradox: traits such as obedience, endurance, and silence are publicly valorized as strength, yet privately experienced as resignation and loss of agency. Participants repeatedly described being praised for their capacity to endure hardship, even as this endurance reinforced expectations of subordination and self-silencing.

Despite the persistence of these social expectations, participants' narratives also revealed signs of disruption and redefinition. Growing public dialogue around mental health, autonomy, and empowerment has opened space for women to challenge these inherited blueprints. For example, a mother emphasized her desire to transmit new forms of strength to her daughter:

“I want my daughter to be strong, but not in the way we were taught; to endure everything in silence. I want her to know it's okay to say no, to choose herself, and to live for her own happiness.” (44 years, married, housewife).

In this reframing, strength was no longer equated with silent endurance but with self-assertion and wellbeing. A young participant echoed this shift, rejecting the notion that strength required emotional suppression. As she stated:

“Being strong doesn't mean hiding your pain anymore. I don't want to pretend everything is fine just to please others. That's not the kind of life I want.” (23 years, single, undergraduate student).

These perspectives illustrate an ongoing cultural transformation in the meanings attached to the SW identity. Whereas traditional blueprints positioned strength as endurance and self-sacrifice, contemporary reinterpretations increasingly align strength with authenticity, self-expression, and emotional openness. This tension between inherited norms and emergent values highlights both the durability of cultural scripts and their capacity for change under evolving social conditions.

#### 3.1.2 Models of a strong woman

Despite growing contestation, the traditional SW identity continues to occupy a position of reverence, anchored in deeply rooted moral and social codes that frame self-sacrifice and caregiving as essential dimensions of womanhood. Within this framework, prioritizing the needs of others above one's own is not perceived as a limitation but rather as a defining marker of strength and virtue. Several participants emphasized that such practices were regarded as inherent to being a “Middle Eastern woman,” reflecting the extent to which caregiving and emotional labor are not viewed as individual choices but as moral obligations that affirm women's social worth. In this sense, endurance and self-denial remain culturally legitimized as the highest expressions of female strength. As a participant explained,

“In our culture, a woman is respected when she puts everyone before herself. It's not considered weakness; it's what makes her a good woman. Taking care of others, even when you're tired, is part of who we are.” (42 years, married, housewife).

The intergenerational transmission of these ideals was particularly salient in participants' accounts, with mothers frequently described as the primary figures who embodied and modeled these values. Through emotional suppression, patience, and personal sacrifice, mothers were portrayed as maintaining family harmony and stability, even at significant personal cost. These lessons were not only observed but deeply internalized. As one participant recalled:

“When I first got married, my mom said, ‘Look at me and your dad. Have you ever heard me fight with your dad?' It means you have to be like your mom… Whether you accept it or not, you are your mom.” (38 years, married, unemployed).

This reflection underscores how maternal example shaped understandings of womanhood, where strength was equated with silence, duty, and endurance.

While many women expressed deep admiration for their mothers' sacrifices, they also acknowledged the emotional costs of this inherited model. This recognition, in some cases, sparked a determination to disrupt the cycle and to cultivate alternative forms of strength, ones that prioritize mental health, self-expression, and self-worth alongside familial responsibility. In this way, inherited ideals remain powerful but are increasingly subject to reinterpretation, as women seek to reconcile cultural reverence for sacrifice with their desire for more sustainable models of womanhood.

Collectively, these narratives reveal the SW identity as a contested and transitional construct in Jordanian cultural life. On one hand, it is idealized as an embodiment of obedience and sacrifice that safeguards family honor. On the other hand, a growing number of women are actively reshaping its meaning, redefining strength to encompass authenticity, agency, and balance. This coexistence of reverence and resistance highlights how inherited ideals both constrain and inspire transformation, sustaining cultural continuity while opening pathways for change across generations.

### 3.2 Women manifesting strength

Participants' narratives highlighted how the notion of the SW is deeply embedded within Jordanian cultural life, shaping women's roles, self-perceptions, and definitions of success. In a context of expanding educational opportunities and increased participation in the workforce, this identity is sustained through pervasive expectations that women should seamlessly balance professional achievement, caregiving responsibilities, and social obligations without outward displays of struggle. Consequently, success is often measured not in terms of personal fulfillment but in the ability to manage multiple responsibilities while maintaining traditional family roles. From early childhood, women internalize this ideal through family and community narratives that rarely invite questioning, reinforcing a cultural script that valorizes resilience, self-sacrifice, and endurance as essential markers of womanhood.

#### 3.2.1 The silent fighter

Participants who embraced the SW identity frequently emphasized patience, endurance, and adaptability as central traits. A participant reflected:

“The SW knows how to handle problems, remains patient, and endures the challenges she faces… A woman may also give up certain things for the sake of something else.” (28 years, single, employed).

Similarly, a participant described the duality of vulnerability and resilience:

“She may cry at night, but in the morning, she wakes up with a plan in mind and a firm decision to move forward.” (46 years, single, employed).

These accounts highlight the complex interplay of emotional vulnerability and strength, where perseverance is valorized as the hallmark of womanhood.

Within the Jordanian context, the SW identity emerged as a cultural archetype admired for her capacity to shoulder expansive responsibilities across domestic and public spheres while maintaining an outward appearance of composure. As one participant noted:

“A SW may be someone who has many responsibilities, whether inside the home, outside, or both; and the ability to coordinate between them while excelling in her life. She must be a fighter, someone who perseveres and overcomes all challenges.” (47 years, married, employed).

The characterization of the SW as a “fighter” positions her not merely as a passive bearer of obligations but as an active agent of survival and achievement. Her strength lies in her ability to negotiate competing demands, suppress personal fatigue, and mobilize inner resources to sustain both family and societal expectations. Yet, this fighter identity is marked by silence: exhaustion and struggle are privately endured, while public narratives celebrate only her resilience and capacity to “move forward.” In this sense, the SW identity reflects both the burdens and the valorization of women's labor, where endurance is reframed as a form of quiet, but relentless combat.

#### 3.2.2 Because she is the giver

Across participants, the SW identity was closely tied to the expectation that women serve as the primary givers of time, care, emotional labor, and stability. Caregiving was described not simply as a task but as a moral imperative embedded in womanhood and reinforced through cultural, religious, and familial norms.

For married and unemployed women, caregiving was portrayed as both an identity and a full-time responsibility that defined their worth within the family. One participant explained:

“My children come to me; they always ask me for everything. They never go to their father because I'm the giver… I'm the one managing everything at home. This is how it is in our Eastern society; inside the home, all the responsibilities fall entirely on the mother.” (38 years, married, unemployed).

For women in this group, caregiving was naturalized as an unavoidable duty that organizes daily life and grounds their social identity.

For married, employed mothers, this belief produced significant strain as they attempted to reconcile professional responsibilities with rigid gender norms at home. One participant described the pressure:

“I'm expected to excel at work and still come home to be the perfect wife and mother. It feels like I'm living two full-time lives at once. And if I fall short in any area, my marriage could be at risk… and then I'm no longer seen as a SW.” (40 years, married, employed).

This quote illustrates how caregiving is moralized as a woman's primary duty, with failure to meet these standards perceived as a threat to her identity and social standing.

Notably, single employed women described facing similar expectations despite not having children. Their narratives demonstrated that the giver role extends beyond motherhood and is applied to women more broadly. As one participant stated:

“Even though I don't have kids, I'm expected to care for my sisters and brothers, cook for the family, and handle emotional issues. That's what women should do.” (46 years, single, employed).

Here, caregiving responsibilities were assigned not because of marital or maternal status but because of gender alone, reinforcing women's position as default sources of both emotional and practical support within extended families.

Participants also emphasized that this giver role is transmitted across generations. One woman reflected on how these expectations were taught from childhood:

“Since I was a little girl, I was told that taking care of others is what makes a woman great. It's what I saw my mother and sisters doing.” (44 years, married, employed).

Such narratives illustrate how nurturing is framed as an intrinsic part of femininity, often superseding professional ambitions and positioning caregiving as a higher calling.

Together, these accounts demonstrate how the giver ideal functions as a pervasive cultural script that shapes women's daily lives across marital, maternal, and employment statuses. By positioning women as the default providers of both emotional and practical labor, these expectations reinforce sacrifice as a feminine virtue while limiting women's autonomy and amplifying the pressures of the SW identity.

#### 3.2.3 Who can fulfill the requirements?

These narratives also underscored the conditional nature of societal recognition. One participant observed,

“I think all superwomen are either single or divorced,” (46 years, married, employed).

Suggesting that autonomy, and with it, the acknowledgment of strength, may be more attainable outside the confines of traditional marital relationships. This perspective highlights how women's capacity to be recognized as “superwomen” is often evaluated through relational status, with independence viewed as affording greater space for self-definition.

At the same time, participants emphasized that strength is not necessarily linked to formal education but can emerge from inner determination and resilience. Several women invoked the examples of their mothers and grandmothers, who, despite lacking formal schooling, managed households, supported their families, and even pursued entrepreneurial ventures. As one participant explained:

“Having degrees isn't necessarily a requirement. Sometimes, a woman simply has a deep desire to learn about everything… Our mothers didn't have formal education or degrees, yet they were capable of handling everything. They managed the home, worked, went outside, and even started their own businesses. They were strong, and their strength came from within.” (45 years, married, employed).

These accounts illustrate how strength and capability are defined in diverse ways, sometimes through autonomy from traditional relationships, and at other times through lived experience, practical knowledge, and inner resolve rather than formal qualifications.

#### 3.2.4 Resisting the identity

While the SW identity was often admired for symbolizing resilience and devotion, many participants also emphasized its heavy psychological toll. The demand to embody unattainable standards of strength and self-sacrifice frequently resulted in exhaustion, self-doubt, and conflict between personal aspirations and cultural obligations. One participant critically reflected:

“Why am I expected to be a SW? I am a person who grows at every stage of my life, and my thoughts are changeable. Who sets these standards for women?” (32 years, married, employed).

Such reflections highlight how gendered cultural scripts are not only internalized but also actively questioned, exposing the tensions between inherited ideals and women's evolving sense of self.

For some participants, the SW role was a source of pride, offering recognition and a sense of purpose. For others, however, it was experienced as an externally imposed narrative that constrained individuality and wellbeing. Women in this latter group openly resisted the identity, rejecting the expectation that endurance and sacrifice should define their worth. In their accounts, the SW figure was reframed not as an aspirational model but as a burdensome cultural script that demanded silence, obedience, and the suppression of authentic needs. As a participant shared:

“They call it strength when you stay silent and accept everything, but that's not strength, it's exhaustion. I've learned that saying no and setting limits is also a kind of strength.” (30 years, single, graduated).

Taken together, these narratives reveal a complex and often ambivalent relationship with the SW identity. While it offers some women a sense of achievement or moral recognition, it simultaneously imposes restrictive expectations that conflict with their evolving needs and aspirations. The growing willingness among participants to question, reinterpret, or openly resist the SW role signals an important cultural shift, one in which traditional ideals of feminine strength are being renegotiated in favor of more balanced, self-sustaining models of womanhood.

### 3.3 Structural barriers and lived struggles

Participants' narratives illuminated how structural and cultural barriers, embedded in family hierarchies, gender norms, and broader societal expectations, profoundly constrained women's autonomy and shaped the course of their lives. From early childhood, many described being socialized into systems where decisions about education, marriage, and career were rarely individual choices but instead negotiated, or imposed, within collective family structures. These accounts underscore how women's life trajectories were often circumscribed by forces beyond their control, situating personal struggles within wider cultural and institutional constraints.

#### 3.3.1 Gendered power and women's autonomy

Participants consistently described how male authority operated as a central force constraining women's choices and autonomy. From an early age, girls were socialized to obey, serve, and self-sacrifice, while boys were encouraged to cultivate leadership, independence, and entitlement to decision-making. These norms were not presented as negotiable but as fixed expectations embedded within patriarchal family structures. As a participant explained:

“We're talking about a patriarchal society, and there's no way for debate. Certain things are simply taken for granted, and we have to admit them.” (58 years, married, employed).

Such accounts reveal how obedience was framed as a marker of female virtue, while authority was reserved for men, limiting women's capacity to envision or pursue alternative futures.

These dynamics were particularly pronounced in rural settings, where fathers, brothers, and male cousins exercised extensive control over women's mobility, education, and marital choices. Many participants linked this authority to Qiwama (male guardianship), which, though religiously grounded, was frequently described as culturally manipulated to justify domination and surveillance. One participant, who grew up in a rural area of the southern region of Jordan, shared:

“When I entered university, I was assigned to study nursing... but I had to specialize in something else... I left the university and went to college so I wouldn't get hurt by my cousin, because of the stigma about nurses.” (48 years, married, employed).

Her narrative illustrates how male authority shaped not only educational opportunities but also women's sense of safety, forcing compliance even when it required abandoning personal aspirations.

These accounts underscore how male authority functioned as both an institutional and relational force, legitimizing control while circumscribing women's agency. Yet within these constraints, women also described subtle acts of defiance, revealing the tensions between imposed compliance and the pursuit of self-determination.

#### 3.3.2 Dual burdens

Women's narratives revealed a persistent struggle to reconcile internal desires with externally imposed expectations; a dual burden produced by the convergence of cultural norms, marital hierarchies, and structural gender inequities. Although many participants were socialized to regard obedience and self-sacrifice as core feminine virtues, their accounts illuminated a continual negotiation between the duty to comply and the growing wish to assert personal agency. This inner conflict often generated feelings of confusion, self-doubt, and emotional fatigue. As a participant described:

“Does the world around you show mercy? Does your environment allow you that peace? It doesn't.” (47 years, married).

For others, this tension became ingrained as an unquestioned pattern of overextension, as reflected by a participant describing her situation of bearing so many responsibilities:

“I got used to the fact that it's always me, that I'm always there. Me, me, me. Twenty-four hours.” (45 years, single, employed).

These accounts demonstrate how the SW identity is internalized as continuous endurance and self-sacrifice, even when such expectations compromise women's wellbeing.

Marriage emerged as a central site where these internal tensions were intensified by structural and relational dynamics. Several women described a discrepancy between public praise for their achievements and the private dismissal or minimization of their efforts by their husbands. One participant noted:

“Even if society sees her as an ideal and successful woman, her husband perceives her differently. To him, her success is not acknowledged.” (47 years, married, employed).

While some women managed to negotiate support over time, such approval was portrayed as conditional and dependent on male authority. As one participant shared regarding her aspirations for postgraduate study:

“At first, he said no… but I explained why it mattered. Now, he tells others he's proud of me and my achievements.” (38 years, married, employed).

Another participant emphasized the centrality of male guardianship (Qiwama) more broadly:

“The first thing I know is that a strong woman always has her father by her side... the father who gives her strength.” (45 years, married, employed).

These reflections highlight how women's legitimacy and recognition remained connected to endorsement from husbands or fathers, reinforcing patriarchal continuity within intimate relationships.

The dual burden was further compounded in professional settings, where women were expected to excel at work while sustaining most domestic responsibilities, yet still faced structural barriers to advancement. Even highly educated women recounted experiences of gendered exclusion. One participant explained:

“I'm given all the opportunities, but by the time I reach the top level… it's always him who gets the final opportunity, simply because he is a man.” (40 years, married, employed).

Others described subtle or overt resistance from male colleagues, particularly when women's success challenged established gender norms. A participant recounted how workplace dynamics shifted after her promotion:

“After I got a higher position, some male colleagues stopped talking to me the same way. One of them said, ‘They just chose you because women are more patient.' It felt like he couldn't accept that I earned it.” (43 years, married).

Such interactions were experienced as direct attempts to undermine women's merit and reinforce male dominance within organizational hierarchies.

Together, these accounts demonstrate how women navigate a paradox of high expectations combined with persistent devaluation. They are required to sustain households and uphold family honor, yet their success remains contingent on male validation and is continuously shadowed by patriarchal norms. The dual burden thus reflects both an internal struggle and an externally imposed system of gendered power that shapes women's everyday lives across personal, relational, and institutional domains.

#### 3.3.3 Constrained choices and aspirations

The cumulative impact of structural and cultural barriers frequently resulted in deferred or unrealized ambitions for women. One participant reflected:

“Sometimes, I wish I could be like a certain person who was able to achieve their goals. But there are people behind me whom I cannot go against... He has been telling me (her husband) for 15 years, ‘I will let you continue your education,' but it still hasn't happened.” (45 years, married, employed).

Her account underscores how opportunities often remain contingent upon male permission, rendering personal aspirations perpetually postponed.

Participants frequently linked these individual experiences to broader systemic issues, highlighting the need not only for women's empowerment but also for cultural transformation that includes men. As a participant stated:

“We need to change the entire system. We need to work on shaping the mindset of future generations; both men and women.” (43 years, married, teacher).

This reflects a recognition that sustainable gender equity requires educating men to support shared decision-making, alongside initiatives to expand women's autonomy.

In sum, these narratives illustrate how structural and cultural barriers operate across domestic, educational, and professional spheres. Women are expected to carry disproportionate responsibilities while recognition, autonomy, and equal opportunity remain circumscribed. Endurance is valorized, yet self-determination remains constrained, highlighting the dual necessity of empowering women and cultivating male partnership to challenge enduring systems of patriarchal authority.

### 3.4 Empowered transitions in women's identity

This theme explores how women described transformative shifts in their identities as they navigated and redefined societal expectations. Participants recounted moments of critical reflection in which they began prioritizing self-care, autonomy, and emotional wellbeing. While some expressed regret over past choices made to conform to external pressures, they simultaneously acknowledged their capacity to redirect their paths. Heightened awareness of mental health and the assertiveness of younger generations were repeatedly identified as catalysts for the emergence of self-determined living.

#### 3.4.1 Cultivating self-belief

Women articulated empowerment as rooted in self-confidence, personal agency, and purposeful action. While traditional depictions of the SW emphasized silent endurance and self-sacrifice, participants increasingly embraced an image of the empowered woman as resilient, ambitious, and guided by her own values. A participant reflected on her journey of studying and working:

“I consider myself a successful woman. Why? Because I pursued my goals and fulfilled my potential. I don't need to be perfect, I took deliberate steps until I reached what I wanted.” (58 years, married, employed).

Similarly, a participant highlighted the integration of aspiration and integrity:

“A SW is someone who sets goals and pursues them with determination, yet she remains grounded in reality and steadfast in her values.” (29 years, single, employed).

These narratives underscore empowerment as active, intentional, and value-driven; demonstrating that women can define success and fulfillment on their own terms rather than conforming to externally imposed ideals.

#### 3.4.2 Strategic self-actualization

Participants framed the SW identity as a pathway to self-actualization, extending empowerment beyond private life into public and professional spheres. Confidence, moral integrity, and meaningful social contribution were central to this process. A participant reflected:

“When I have confidence in myself and my abilities, I become a super person. My values and morals give me inner strength. I contribute, I produce, and I become a positive force in society.” (40 years, single, employed).

In this perspective, true influence stems from purposeful engagement and meaningful participation rather than external recognition.

Key strategies for self-actualization included setting boundaries and prioritizing personal needs. A participant emphasized:

“To be a SW, I have to prioritize myself. I have to truly know and honor my own needs.” (28 years, single, employed).

Equally important was the pursuit of balance; integrating ambition with emotional wellbeing through reflection, self-awareness, and resilience. As a participant explained:

“Through the ups and downs, and the difficult circumstances in my life, I have learned that a person must cultivate inner peace, understand their strengths and weaknesses, and embrace themselves fully.” (42 years, single, employed).

These narratives portray self-actualization as an intentional, ongoing journey in which women assert autonomy, cultivate inner growth, and create meaningful impact in the world.

#### 3.4.3 Financial and intellectual autonomy

Participants consistently highlighted independence, both financial and intellectual, as a core dimension of empowerment. Financial self-sufficiency was described not merely as a personal achievement but as a crucial foundation for agency and self-determination. A participant stated:

“To me, a SW is an independent woman who can fully support herself. I consider myself a SW when I am 100% financially independent and when I think and make decisions freely.” (40 years, single, employed).

Another participant emphasized the importance of intellectual autonomy, explaining:

“For me, strength is also in the mind. When I have the knowledge and the confidence to form my own opinions, I don't let anyone decide for me. Thinking for myself is part of being a strong woman.” (35 years, married, employed).

These perspectives frame financial and intellectual autonomy as a gateway to negotiating social expectations on one's own terms, providing women the freedom to pursue their ambitions, set boundaries, and engage in society without reliance on others. Intellectual independence complemented this, enabling women to make informed choices and assert their perspectives, reinforcing a holistic model of empowerment grounded in self-reliance and agency.

#### 3.4.4 Pursuing wholeness

Empowerment was further conceptualized as holistic, integrating personal, spiritual, and emotional dimensions. Women emphasized alignment between values, goals, and daily practice as central to achieving inner balance and fulfillment. A participant explained:

“At every stage of your life, you have a goal… and you reach inner peace, whether you are a housewife or in your profession. When you reach this goal… inner peace and financial independence are the most important things.” (39 years, married, employed).

Spirituality also provided moral orientation and motivation. A participant said:

“When we are in a difficult situation, and we endure and sacrifice, we say, ‘That's it, I will be in heaven (Jannah), better than anything.' This is a motivation.” (44 years, married, housewife).

These narratives situate empowerment not solely in external achievements but in the cultivation of wholeness, an integrated sense of self grounded in purpose, resilience, and ethical clarity.

Collectively, these accounts illustrate the multifaceted ways Jordanian women conceptualize empowerment and success. For many, the SW identity has evolved beyond silent sacrifice, redefined through intentionality, independence, and resilience. Strength is reframed as pursuing self-defined goals, cultivating inner balance, and asserting personal values while navigating social expectations. Yet for others, the SW remains a contested construct, anchored in traditional ideals of endurance, but gradually reimagined as a figure of agency, authenticity, and principled self-determination within a shifting cultural landscape.

### 3.5 Everyday coping and sustainable wellbeing

This theme explores the strategies Jordanian women employed to manage stress and cultivate mental wellbeing, highlighting a shift from passive endurance to intentional self-preservation. Participants described a range of practices that fostered resilience, encompassing relational, spiritual, and embodied approaches, as well as deliberate acts of self-care.

#### 3.5.1 Relational anchors

For many participants, resilience was not rooted in solitary endurance but in the support, reciprocity, and shared labor. Women described how distributing tasks, seeking assistance, and engaging in mutual aid across family, workplace, and community contexts strengthened both their sense of autonomy and their social bonds. One participant reflected on this shift:

“I used to think I had to do everything myself to prove I was strong. But now I see that asking for help doesn't make me weaker; it actually gives me more strength.” (42 years, married, employed).

Another participant highlighted the emotional relief found in interpersonal connection:

“When I talk to my sisters or close friends, I feel my load becoming lighter. Just sharing what's in my heart makes me stronger than carrying everything alone.” (39 years, married, employed).

These narratives challenge cultural scripts that equate strength with silent endurance. Instead, women framed relational connection as a vital resource that fosters empowerment, reduces isolation, and creates collective pathways for coping with daily pressures.

#### 3.5.2 Grounding practices

Participants emphasized the importance of intentional practices that allowed them to pause, restore energy, and sustain emotional balance. Everyday routines (journaling, reading, exercising, or taking brief moments of solitude), were described as deliberate strategies for self-regulation rather than indulgences. As one woman explained:

“I exercise. I go to cut my hair just like that, any form of happiness. Simple things… can improve our mental health. These things give us energy and happiness as well.” (28 years, single, employed),

another said:

“When things become overwhelming, I write. It helps me sort my thoughts and feel supported, even if it's only by my own words on the page.” (32 years, single, employed).

Spirituality played a central role in these grounding practices. Women highlighted prayer, reflection, and worship as essential sources of inner stability. One participant stated:

“As long as I fulfill my duties in this world for the sake of Allah (God)… everything else will fall into place. A person must balance their life and remember they have a right to take care of themselves through prayer and worship to recharge and stay grounded.” (45 years, married, unemployed).

Another described taking brief daily pauses:

“I make it a point to pause each day, even for a few minutes, to reflect, pray, or simply breathe. It helps to handle my responsibilities without losing myself in the process.” (35 years, married, graduated).

Across narratives, intentional care emerged as a multidimensional approach that nurtured clarity, resilience, and a sense of moral and emotional alignment.

#### 3.5.3 Joyful engagement

Participants frequently described coping not only as managing stress but as cultivating joy, creativity, and embodied presence in daily life. Engaging in activities such as handicrafts, travel, or visiting loved ones was seen as a proactive way to reaffirm self-worth and renew emotional energy. One participant shared her experience in recycling:

“When I create something and accomplish it… my entire day feels positive. It reflects on my home, my children, and even on any guest who visits.” (43 years, married, unemployed).

Another highlighted the restorative power of exploration and social connection:

“I also find joy in visiting loved ones, exploring new places, or even buying something special during their trips.” (30 years, single, employed).

Physical engagement was also portrayed as essential for emotional regulation and mental clarity. As a participant noted:

“Walking, exercise, shopping... these things can improve my mood.” (33 years, married, graduated).

Another said:

“Taking a walk, doing some exercise, or even running errands gives me a break from my worries and helps me think more clearly. It's like pressing a reset button for my mind.” (44 years, married, employed).

These accounts demonstrate that embodied and joyful activities serve not only as coping mechanisms but as vital forms of self-affirmation that counterbalance the pressures of daily life. This subtheme focuses on activities that energize, encourage, and refresh women's sense of self through positive involvement with the world, compared to the previous subtheme's emphasis on practices that steady and recharge women from inside.

Together, these narratives reveal a holistic model of sustainable wellbeing grounded in connection, intentional self-care, and joy. Women's accounts demonstrate a shift away from the traditional expectation of silent self-sacrifice toward more balanced and life-affirming practices. In redefining resilience through relational support, spiritual grounding, and meaningful engagement, participants articulated a contemporary vision of the Jordanian SW, one who integrates care for others with care for herself, and who cultivates strength not through exhaustion, but through connection, purpose, and renewed vitality.

## 4 Discussion

The findings illustrate that Jordanian women define the traditional SW identity through ideals of silent endurance, self-sacrifice, and unconditional caregiving, reflecting societal norms that position men as financial providers while women shoulder emotional and domestic labor. Within this context, the SW identity is widely perceived as a moral expectation rather than a personal choice, often carrying significant emotional weight. Women expressed mixed responses to these expectations, some internalizing them as sources of pride and duty, while others described frustration, exhaustion, or a desire to resist them. Increased exposure to social media, mental-health discourse, and global narratives of empowerment has reshaped how many women conceptualize strength, creating tension between evolving desires for autonomy and longstanding obligations of obedience and resilience. Notably, several participants expressed hopes for their daughters to pursue lives grounded in self-care and agency, highlighting a growing generational shift in the meaning and desirability of the SW role.

When situated within the Western SWS literature (Woods-Giscombé, 2010; Beauboeuf-Lafontant, 2009), these patterns reveal both continuity and divergence: although the core SWS dimensions (strength, emotional suppression, resistance to vulnerability, perseverance under limited resources, and prioritization of caregiving) are evident in Jordan, they are shaped by culturally specific frameworks that emphasize moral virtue, familial duty, and gendered expectations rather than the racialized histories highlighted in Western research. Notably, many women also resist or reinterpret these norms, seeking autonomy, education, and emotional authenticity while navigating the constraints of traditional expectations, illustrating that their struggles reflect broader structural and cultural forces rather than solely individual psychological challenges.

Findings from the focus group discussions demonstrate that in this patriarchal and religiously conservative context, societal narratives prioritize women who can seamlessly balance familial and social responsibilities. Unlike the Western context that emphasizes the shift toward paid employment as part of a gender-role revolution for women (Genz and Genz, 2009), having a paid job was viewed as secondary, as financial provision is primarily considered a male responsibility. Consistent with Western research (Leath et al., 2023), Jordanian women described learning this identity early in life through maternal modeling and communal reinforcement. However, in this context (and particularly following exposure to Western ideals of strength and independence) the defining traits of obedience and caregiving (especially motherhood) emerged as significant sites of internal challenge, underscoring how the SWS is fundamentally reshaped by Middle Eastern sociocultural and religious traditions (Koburtay et al., 2023; Aljaouhari, 2014).

This study confirmed that the SW identity is transmitted and reinforced through cultural narratives and intergenerational modeling. This ambivalence reflects a central paradox: the very attributes that are celebrated, strength, perseverance, and emotional restraint, can simultaneously erode wellbeing by normalizing self-sacrifice and discouraging vulnerability. These findings resonate with Western research (Woods-Giscombé, 2010; Beauboeuf-Lafontant, 2009; Harris-Lacewell, 2001; Erving et al., 2024a; Nelson et al., 2022, which similarly documents how the ideal of strength can foster motivation and purpose, yet emotional control often intensifies stress, isolation, and psychological distress.

Importantly, participants' responses to the SW ideal varied significantly. The findings suggest a spectrum of engagement, ranging from outright rejection of the identity's constraints and excessive burdens (“Why am I expected to be a SW?”), to the purposeful adoption of the identity as a source of perseverance and social belonging (“I consider myself a SW, yet my society does not perceive me as such…”). This ambivalence reflects the dual function of the SWS; it can serve as a coping resource that fosters resilience, yet also operates as a mechanism of gendered control that legitimizes women's subordination (Nelson et al., 2016; McDaniel et al., 2023; Woods-Giscombé, 2010). In the Jordanian context, identity is not merely internalized but actively reproduced through cultural norms, religious interpretations, and intergenerational socialization (Koburtay et al., 2023). Rooted in patriarchal expectations (Ebert, 1988; Barragan et al., 2018), the ideal of the “strong woman” is framed less as empowerment than as self-sacrifice and emotional endurance. Obedience, in particular, emerges as a salient marker of moral worth, binding women's sense of strength to compliance with religious and cultural prescriptions (Aljaouhari, 2014; Franks and Franks, 2001). Thus, what appears as an affirmation of women's resilience simultaneously functions to naturalize structural inequalities and constrain possibilities for autonomy.

Caregiving, especially motherhood, was central to participants' self-definition, often overriding personal aspirations or career advancement. This is consistent with prior findings (Bugden et al., 2025; Bullock and Waugh, 2004; Aston, 2000). Even childless women reported carrying substantial caregiving burdens, reflecting how womanhood itself is equated with selfless service to others, consistent with prior findings (Araci Iyiaydin and Ergun, 2025; Lee, 2025; Abrams et al., 2014). These expectations contribute to invisible but taxing emotional labor (Beauboeuf-Lafontant, 2009; Donovan and West, 2015; Watson and Hunter, 2015; Shah et al., 2025).

Socioeconomic position and marital status also shaped how women negotiated this identity. Single and divorced women described greater autonomy and opportunity to pursue long-deferred aspirations, echoing research on gender roles in patriarchal societies (Almog and Herbst-Debby, 2025; Erving et al., 2024a,b). In contrast, married women often encountered male-dominated decision-making structures, reinforced by restrictive interpretations of Qiwama (male guardianship), which undermined women's self-determination (Abou-Bakr, 2015; Labib, 2023).

The dual burden of professional and domestic labor emerged as a persistent source of tension. Women were expected not only to excel in the workplace but also to remain the primary caregivers at home, reproducing entrenched patterns of gendered multitasking (Offer and Schneider, 2011; Rose, 2017). Even at the peak of professional achievement, they were positioned as default caretakers, a role that generated chronic stress and emotional exhaustion (Broadley, 2021). Several participants reported that their professional success provoked unease among male counterparts, prompting subtle or overt attempts to minimize their accomplishments. A minority, however, described spousal support as a protective factor (Prasiska et al., 2024; Matsui et al., 1995). Such support remains the exception in Middle Eastern patriarchal contexts, where men are still framed as economic providers rather than domestic contributors. Within this structure, the SW identity functions less as a freely chosen ideal than as a cultural mandate that legitimizes unequal divisions of labor. It offers women recognition through strength and endurance, yet simultaneously constrains them by naturalizing sacrifice and positioning caregiving as their primary obligation. This paradox underscores the mental health costs of idealized feminine roles and the structural inequities that sustain them (Parks and Hayman, 2024).

Maternal figures played a central role in transmitting the SW ideal. Women recalled being raised to admire mothers who embodied silent resilience, emotional suppression, and sacrifice, models that were both revered and exhausting. This mirrors earlier work on maternal modeling of sacrifice (Bainbridge et al., 2021; Brémault-Phillips et al., 2016; Kramer, 2005) and highlights the psychological costs of internalizing restrictive feminine ideals (Hoffmann and Mitchell, 1998). At the same time, many younger women in this study rejected the notion that resilience requires self-denial. Instead, they emphasized emotional authenticity, boundary-setting, and self-care as essential markers of empowered womanhood. This reorientation reflects a culturally situated but transformative redefinition of gender roles, resonating with global feminist calls to move beyond sacrifice as the foundation of empowerment (Hay, 2017).

As outlined in the introduction, the adapted SWS framework situates women's strength within moral and religious expectations of patience (ṣabr) and self-sacrifice. The present findings confirm that these culturally embedded virtues continue to structure women's sense of worth, even as younger generations reinterpret strength through emotional openness and self-care. While the original SWS model emerged in a Western, racialized, and individualistic context, this study demonstrates how Jordanian women re-signify the SW identity through collectivist and faith-based ideals of moral endurance rather than autonomy. This cross-cultural reinterpretation underscores the need for contextual sensitivity when applying Western frameworks to non-Western societies.

Despite these shifts, the SW identity continues to be invoked to justify women's unrelenting availability and endurance, especially in rural and conservative contexts. This underscores the need not only for individual empowerment but also for structural change in how gender expectations are socialized, legitimized, and institutionalized. Participants also described developing coping strategies that resisted solitary sacrifice in favor of relational and spiritual resilience. Seeking help from family, friends, or faith was reframed as a strength, countering dominant stigmas around help-seeking observed across Arab and global contexts (Corrigan et al., 2014; Cheng et al., 2013; Alluhaibi and Awadalla, 2022). Spirituality emerged as a consistent grounding force, providing both emotional stability and moral meaning. These narratives support a redefinition of the SW ideal, not as silent endurance, but as conscious, relational, and holistic resilience.

As a summary, from the lens of Jordanian society, the SW is the one who can endure everything in silence, and she is the giver and the one who should bear all the responsibilities inside or outside the home, because the men he is the financial provider. But after the widespread use of social media and the increased awareness of mental health and women's empowerment, the women's definition of being a SW or being strong has changed, but now they are struggling to balance the traditional and modern ideas of being SW. finally, several women admitted that they don't want their daughters to live the same life as them, under the societal pressure of traditional life in this modern world, and they encourage them to find themselves and take care of it.

Overall, the findings demonstrate that Jordanian women's experiences of strength are shaped by sociocultural expectations of obedience, self-sacrifice, and caregiving, reinforced through religious values, patriarchal family structures, and generational norms that position women as the emotional and domestic anchors of the household. As they navigate these overlapping responsibilities, women internalize competing demands for resilience, silence, and moral duty, often resulting in emotional strain and ongoing negotiation between personal aspirations and societal obligations.

Cumulatively, these findings extend SWS theory by illuminating its reconfiguration within a Middle Eastern context. By highlighting both the convergences and divergences between the Western and Middle Eastern manifestations of the SW ideal, this study contributes to a more inclusive cross-cultural understanding of strength, caregiving, and emotional labor. Future research could build on these insights by developing and validating an indigenized SWS model for Arab societies, one that better captures the moral, religious, and collectivist dimensions of women's lived experiences.

### 4.1 Implications

These findings underscore the need for multi-level action. Structurally, policies must prioritize women's wellbeing by promoting shared caregiving in workplaces, integrating gender equity and mental-health literacy into schools, and challenging the cultural normalization of women's silent sacrifice through public campaigns. Partnerships with religious and community leaders are vital to counter restrictive interpretations of Qiwama (male guardianship), and to promote narratives affirming women's dignity and autonomy. Expanding affordable, community-based mental health services, especially in rural areas, would further dismantle barriers to care.

Culturally, however, the challenge extends beyond the provision of resources to the transformation of social values that normalize women's self-sacrifice and endurance as moral expectations. Sustainable change requires engaging the counterparts of patriarchal systems (men, elders, and influential community figures) in awareness and education initiatives that encourage shared responsibility for emotional and domestic labor. Without such shifts in belief systems, policy and clinical interventions may remain limited in impact.

Clinically, interventions should be culturally responsive, recognizing that socially valorized traits such as endurance and self-sacrifice may conceal distress. Therapists should validate spiritual and relational coping while fostering boundary-setting, self-compassion, and emotional authenticity as legitimate resilience strategies. Group-based, culturally adapted cognitive-behavioral and psychoeducational programs hold particular promise for reducing stigma, normalizing help-seeking, and reframing self-care as both permissible and necessary. Faith-based resources, when integrated critically, can empower rather than constrain.

Finally, future research must address men's perspectives on the SW identity in Islamic societies. Understanding whether women's agency is perceived as threatening or complementary to traditional norms is essential for designing more comprehensive policies and interventions. By linking structural reform, culturally sensitive practice, and attention to gendered perceptions, sustainable conditions can be fostered in which women exercise agency, balance multiple roles, and safeguard their wellbeing.

### 4.2 Limitations

While this study provides important insights into how Jordanian women internalize and navigate the SWS, several limitations shape the scope of its conclusions. The relatively small, gender-specific sample, though recruited from diverse regions, underplays potential sampling bias and may not fully capture the heterogeneity of women's experiences across socio-economic, educational, or religious groups. As such, the findings should be interpreted as illustrative rather than generalizable. While the exclusive focus on women's narratives aligns with the central aims of this study, it inherently excludes the perspectives of men, family members, and community figures whose views and practices play a significant role in shaping gender norms and the construction of the SWS. In addition, although efforts were made to minimize the moderator influence, the role of the moderator may have exerted some effect on the discussions; however, the moderator was specifically trained to maintain neutrality and reduce potential bias, and this limitation is considered minor relative to the rich, nuanced insights gained through this method.

A key point of this study lies in applying the SWS framework (originally conceptualized within a Western, individualistic context and rooted in the experiences of African American women navigating racialized and gendered stress) to a Middle Eastern, collectivist society. While Woods-Giscombe's model provides a valuable lens for understanding the interplay between strength, emotional suppression, and caregiving, its sociocultural origins differ from the context in which Jordanian women construct meaning around these roles. Participants in this study interpreted the “Superwoman” identity through local cultural and religious discourses emphasizing obedience, sacrifice, and familial duty, which both converge with and diverge from the Western notion of independence and self-reliance. These contextual differences should be considered, and future research may benefit from developing an indigenized framework that better reflects the cultural nuances of women's lived experiences in Arab societies.

Methodological considerations further temper the conclusions. The use of focus groups, although effective for generating discussion, may have encouraged social desirability bias, particularly given the cultural sensitivity surrounding gender roles, caregiving, and emotional disclosure. Complementary methods, such as in-depth interviews or extended ethnographic immersion, could help capture more private and counter-narrative dimensions of identity negotiation. Finally, as the study involved translation of interviews from Arabic into English, subtle shifts in meaning and nuance may have occurred despite efforts to maintain accuracy. Taken together, these limitations highlight the need for broader, more inclusive, and methodologically varied approaches to fully apprehend the evolving contours of the SWS in Jordan.

## 5 Conclusion

The SWS among Jordanian women reveals a deep conflict between traditional and evolving interpretations of what it means to be a “strong” woman in a conservative, patriarchal society. For many, strength is idealized through obedience, sacrifice, and resilience, roles that provide social validation but often come at the expense of emotional expression and personal fulfillment. Others, however, described this ideal as a source of psychological strain, linking the constant pressure to endure with feelings of exhaustion, suppressed emotions, and diminished autonomy. This tension illustrates how the same cultural ideal can simultaneously function as a source of pride and as a mechanism of self-silencing and distress. At the same time, the concept of the SW is actively being redefined. Younger participants, in particular, framed strength not only as perseverance but also as self-care, setting boundaries, and embracing emotional openness. These shifting interpretations suggest an ongoing cultural evolution in which endurance remains valued, yet is increasingly supplemented or even challenged by more balanced and sustainable models of womanhood.

By highlighting both the contradictions and the transformations within the SW identity, this study extends the framework beyond its Western origins to show how women in conservative, patriarchal contexts negotiate competing expectations. Future research should examine how these conflicts and evolving meanings are influenced by family and community dynamics, as well as test culturally adapted interventions that encourage help-seeking and validate self-care as integral to women's empowerment. As these narratives demonstrate, empowerment emerges not from carrying every burden, but from the freedom to define strength in ways that sustain both collective responsibilities and individual wellbeing.

## Data Availability

The original contributions presented in the study are included in the article/Supplementary material, further inquiries can be directed to the corresponding author.

## Footnotes

1 Jordan Department of Statistics (2024). Available online at: https://dosweb.dos.gov.jo/.
