# Supplementary material for: Cultural and psychological dimensions of the superwoman identity among Jordanian women
Source: Front Psychol. 2026 Feb 18;17:1676125. doi: 10.3389/fpsyg.2026.1676125 (PMC12956632; doi:10.3389/fpsyg.2026.1676125)
Supplement: Supplementary file 1 [file Table_1.docx]

## Focus group discussion questions

The focus group discussions were guided by the following open-ended questions:

1. What comes to your mind when you hear the term "Superwoman"?
2. For you, what does it mean to be Superwoman?
3. Is there someone you know or have seen who you think represents what it means to be a Superwoman? Why do you view her that way?
4. What do you think society expects from a woman who is seen as a Superwoman?
5. What kinds of emotions do you think a Superwoman typically experiences because of the roles and expectations she carries?
6. How easy or difficult is it for a Superwoman to ask for help when she is overwhelmed? Can you explain why?
7. In your opinion, what helps a Superwoman stay balanced and emotionally healthy despite all the responsibilities on her?
